# Learning and Memory Recoveries in a Young Girl Treated with Growth Hormone and Neurorehabilitation

**DOI:** 10.3390/jcm5020014

**Published:** 2016-01-26

**Authors:** Jesús Devesa, Hortensia Lema, Eva Zas, Borja Munín, Pilar Taboada, Pablo Devesa

**Affiliations:** 1 Medical Centre Foltra, Travesía de Montouto 24, Teo 15886, Spain; hortpeque@hotmail.com (H.L.); evazasm@gmail.com (E.Z.); borjamunin@gmail.com (B.M.); taboada_pernas@hotmail.com (P.T.); pdevesap@foltra.org (P.D.); 2 Department of Physiology, School of Medicine, University of Santiago de Compostela, Santiago de Compostela 15710, Spain

**Keywords:** growth hormone, neurorehabilitation, brain plasticity, cognitive functions, memory, natal asphyxia

## Abstract

Background—To describe the results obtained after treating a non growth hormone-deficient 10-year-old girl who suffered asphyxia during delivery, resulting in important cognitive deficits, with growth hormone (GH) and neurorehabilitation. Methods—GH was administered (mg/day) at doses of 0.5 over three months followed by 0.9, every two weeks over three months, and then alternating 1.2 three days/week and 0.3 two days/week. Neurorehabilitation consisted of daily sessions of neurostimulation, speech therapy, occupational therapy and auditive stimulation. Treatment lasted nine months. Results—Scores obtained in all the areas treated showed that, at discharge, the patient clearly increased her cognitive abilities, memory and language competence index; her intelligence quotient score increased from 51 to 80, and the index of functional independence measure reached a value of 120 over 126 (maximal value). Conclusions—This case suggests that GH administration may play a role in improving cognitive deficits during neurorehabilitation in children with brain damage suffered during delivery. This agrees with the known effects of GH on cognition.

## 1. Introduction

Perinatal Asphyxia (PA) or Hypoxia-Ischemia (HI) are a major pediatric issues, usually occurring when delivery is prolonged, with few successful therapies to prevent neuronal damage [1]. In Western countries they affect two to six infants in every 1000 live born children [2].

Severe asphyxia has been linked to cerebral palsy (CP), mental retardation, and epilepsy [3,4,5], while mild-moderate asphyxia has been associated with cognitive and behavioral alterations, such as hyperactivity, autism [4], attention deficits in children and adolescents [6,7], low intelligence quotient score (IQ) [8], and development of psychotic disorders in adulthood [9]. It has been suggested that the decrease in neurotrophic factors induced by PA/HI may lead to dendritic atrophy and disruption of synaptogenesis, effects which are present in individuals destined to develop schizophrenia as adults [10]. PA/HI were previously considered the major cause of CP, a catastrophic acquired disease occurring during the development of the fetal or infant brain. It mainly affects the motor control centers of the developing brain, but can also affect cognitive functions, and it is usually accompanied by a cohort of symptoms including lack of communication, epilepsy, and alterations in behavior [11]. However, current knowledge suggests that prenatal causes are most important [12].

There is consensus among specialists about the need for early neurorehabilitation to improve the natural mechanisms involved in brain repair after an injury and to achieve the best possible functional and social recovery [13]. However, access to rehabilitation facilities specializing in early care in children born with problems is marked by the shortage of public or private resources, with a huge difference in the availability of such facilities among the different countries. This shortage, scarcity or nonexistence of public or private rehabilitation facilities in many countries, together with the high cost of these services offered by private centers, make access to rehabilitation difficult or even impossible for many people. Moreover, apart from the need for an early, intense and specific rehabilitation, medical science has little to offer for the persistent symptoms that prevent many of these children from partially or fully living a normal life.

Years ago, a surprisingly high prevalence of GH-deficiency (GHD) had been described in a small population of CP children [14]. More recent studies confirmed those findings in a larger number of CP children [11,15]. GH treatment, together with neurorehabilitation, has been reported to induce beneficial effects on motor and cognitive functions in GHD CP children [16,17], but also in non GHD patients who had suffered a very significant traumatic brain injury [18,19].

The GH receptor is expressed in regions of the brain in which neurogenesis occurs during embryonic brain development [20,21], as well as in neurogenic regions of the postnatal rat brain [22]. Growth hormone itself is also found in cells of the ventricular zone during embryonic neurogenesis [21], and is produced endogenously within the postnatal hippocampus [23,24]. In fact, the GH/Insulin-like growth factor 1 (IGF-I) axis plays a pivotal role during human fetal brain development and a very important role in cognitive functions afterwards [25]. Moreover, GH may facilitate brain plasticity [26]; the hormone induces the expression of a number of neurotrophic factors (IGF-I, Epidermal growth factor (EGF) and its receptor, Basic fibroblast growth factor (FGF-2) Erythropoietin (EPO), Vascular endothelial growth factor (VEGF), Brain-derived neurotrophic factor (BDNF)) and increases the cerebral metabolic turnover of noradrenaline (NA) and dopamine (DA) [27].

This report describes the results obtained after treating a non-GHD young girl who suffered asphyxia during delivery leading her to important cognitive deficits, without any motor affectation with GH and neurorehabilitation.

## 2. Experimental Section

### 2.1. Patient

The patient was a 10-year-old girl who was born, at week 41, by emergency cesarean due to fetal sustained bradicardia once labor work had begun without progression. She presented an umbilical cord tight around the neck. Apgar score after birth was 0-3-6, needing reanimation and assisted ventilation. Sixteen hours later, the patient began to develop repeated tonic-clonic movements, pharmacologically uncontrolled, accompanied by hypertony, apnea and cyanosis. A brain ultrasonography revealed the existence of a right subependymal hemorrhage.

Routine studies carried out for neonatal screening for hereditary diseases were normal (*i.e.*, congenital hypothyroidism, diseases detected by tandem mass spectrometry, *etc*.). Since the patient evolved favorably, 20 days after birth she was discharged.

However, during her development a number of anomalies related to cognitive functions were appearing: low intelligence quotient score (IQ), loss of recent memory, attentional deficits, specific language disorder (anomic-syntactic syndrome). These deficits had been treated, in different centers, with neurorehabilitation (speech therapy and neurostimulation) and medication (oral methylphenidate hydrochloride, 5 mg/day, Concerta, Janssen; later, 10 mg/day, Medikinet, Rovi). According to the therapy reports provided by the family, speech therapy and neurostimulation previously performed were similar in procedures and intensity to those that the patient received at our center. Assessment scales for the evaluation of these therapies were also similar to those we used (Peabody test and Boston test for speech therapies, and the Wechsler Intelligence Scale for Children (WISC-IV) test (at the age of seven years) for neurostimulation). No significant evolution was found during these years where neurorehabilitation was attempted.

At the age of 10 years, a brain MRI indicated the existence of areas of encephalomalacia in frontal lobes, marked cortical atrophy, limbic system alterations, decreased white matter thickness and focal thinning of the corpus callosum. Both hippocampi were normal.

An electrophysiological study (auditive evoked potentials (PEAT)) detected the existence of mild bilateral hypoacusia.

At admission, clinical examination indicated that height and weight were normal for her age (p50 in both cases), with no motor problems, but lack of comprehension, lack of the language appropriate to her age (according to the mother the patient had been unable to say short phrases until age of seven years), and a clear loss of recent memory (she was unable to remember anything happening a few minutes earlier). In summary, the main problems were: alteration of the cognitive functions for maintaining attention, expressive and receptive speech, verbal declarative memory, low processing speed and a compulsive behavior. Routine blood analyses were normal and no hormonal pituitary deficits existed (plasma levels of TSH, fT4, cortisol, IGF-I; FSH and LH were in the prepubertal range). A study of GH responses to provocative stimuli was not performed given the normal growth velocity of the patient, her height and the normal plasma levels of IGF-I and IGFBP3.

### 2.2. Treatments

After obtaining signed informed consent, the patient was scheduled for GH treatment (Nutropín, Ipsen; 0.5 mg/day, sc, five days/week over three months followed by 0.9 mg/day, five days/week, every two weeks during three months and then alternating 1.2 mg/day, three days week, and 0.3 mg/day, two days/week, until the end of the treatment). The rationale for this was based on our previous studies indicating that high doses of GH favor brain stem cell differentiation, while low doses favor the proliferation of these neural progenitor cells [28]. Therapies for neurorehabilitation consisted of: neurostimulation, speech therapy, occupational therapy and auditive stimulation (similar to that described by Tomatis [29]). Every stimulation session (five days per week) lasted 45 min, except for auditive stimulation (30 min per day, three days per week). These therapies were maintained throughout the whole treatment, whether or not GH was administered. Studies and treatments were conducted in compliance with national legislation and the Code of Ethics of the World Medical Association (Declaration of Helsinki).

Clinical assessments and blood analysis (including thyroid hormones, IGF-I and IGFBP3), were performed every three months. Therapy assessments (standard tests) were performed at admission and at the end of the treatment, before discharge (nine months after admission). None of the therapists knew the medical treatment the patient was receiving at any moment.

## 3. Results

### 3.1. Clinical Assessments

Clinical exams at admission did not show any physical or motor abnormality. Cardiac auscultation was normal; cranial nerves, muscle tone and tendon reflexes were normal; gait and equilibrium were normal and fine motor functions were also normal. There was a mild bilateral hypoacusia and significant cognitive deficits. The patient had a marked attention deficit and compulsive behavior. Her height was 138 cm (p50) and her weight was 30.800 kg (p50). The Tanner stage of pubertal development was 4. At discharge, no significant changes in pubertal development were observed while her height had increased to 143 cm (p62) and her weight to 32.500 kg (p55).

Table 1 shows the results of the different therapies’ assessments at admission and before discharge.

**Table 1:** Scores obtained in the different areas of stimulation, at admission (Pre-) and before discharge (Post-). Neurostimulation (WISC-IV test): *VC* = verbal comprehension; *PR* = perceptual reasoning; *WM* = working memory; *PS* = processing speed; *IQ* = intelligence quotient score. Speech therapy (Peabody and Boston test for aphasias). *EA* = Equivalent age (years.months), *p* = percentile; Occupational therapy (WeeFIM test for Functional index measure): *PC* = personal care; *Mo* = mobility; *Me* = memory; *SP* = ability for solving problems. In the WeeFIM test only some significant results are showed.

**WISC-IV**	**Pre-**	**Post-**
*VC*	13	18
*PR*	14	23
*WM*	12	21
*PS*	12	18
*IQ*	51	80
**Peabody**	**Pre-**	**Post-**
*EA*	6.0	8.11
*IQ*	55	75
*p*	0.1	2
**Boston**	**Pre-**	**Post-**
*Aphasia*	40.1	55
**WeeFIM**	**Pre-**	**Post-**
*PC*	50	55
*Mo*	35	35
*Me*	1	5
*SP*	2	6

### 3.2. Neurostimulation

At admission, the WISC-IV test, a scale for measuring the intelligence in children, reached a score of 51, clearly indicative of a very low IQ for the age of the patient. This score resulted from the low punctuations reached in: verbal comprehension, index of perceptual reasoning, working memory and processing speed. On this basis, we started working through a systematic training in tasks of language, memory and attention, especially in areas related to solving tasks in nonverbal content; we also established guidelines for generalization of training to everyday contexts and searched for motivating elements, and we also used visual tasks with some verbal content, working with close, everyday objects, providing memory strategies, simple verbal labels and marking her time to complete them. We also focused on attention. The difficulty of the tasks proposed was increasing as the patient was improving. Therefore, results obtained in the WISC-IV test, particularly in perceptual reasoning and working memory, significantly increased until a value of 80 was reached at the end of the treatment period. This value indicates that the patient improved her IQ to the lower limit of normality for her age. The behavior of the patient inside and outside the Centre (easy frustration, attentional deficit, compulsive behavior) considerably improved too, agreeing with the results obtained in the WISC-IV test.

### 3.3. Speech Therapy

At admission, the patient presented a specific language disorder, dyslexic traits, difficulties in acquiring concepts of laterality and body schema, a deficit in episodic memory and anomie. The Peabody test indicated that the age of the patient would be equivalent to that of a six-year-old child and her IQ was 55, percentile 0.1. These scores increased at the end of the treatment, then reaching the equivalent of a child of eight years and 11 months (in terms of vocabulary), where the IQ was now 75 and the percentile increased to 2.

On the other hand, the Boston test for aphasia and related disorders showed that while the initial language competence index was 40.1, clearly under the media (50), it increased to a value of 55, which is a normal value.

### 3.4. Occupational Therapy

Assessments in this area were carried out by using the WeeFIM test (functional independence measure). At admission, the subtest measuring the degree of personal care had a score of 50 (maximal independence: 56), since the patient only had problems at the time of cleaning herself without the help of another person and was unable to tie her shoelaces. These improved, reaching a final score of 55.

No problems existed in the area of mobility: 35 points at admission and at discharge (total independence).

In the area related to cognition, the patient improved from 18 over 35 to 31 over 35. The highest evolution was observed in the fields of understanding, verbal expression, and memory and problem resolution. There was marked improvement particularly in these two last aspects, since the score for memory was 1 at admission (maximal dependence) and 5 at discharge, while the score in solving problems evolved from 2 to 6. In all, the final WeeFIM score of the patient was 120 (maximal independence = 126).

### 3.5. Auditive Stimulation

Auditive stimulation was designed for increasing the self-motivation of the patient to listen, to correct audio-vocal control and to tune the circuit between the ear and the voice, something very important for her own control of speech, according to the descriptions of Tomatís [29] and modified by our group.

**Figure 1:**
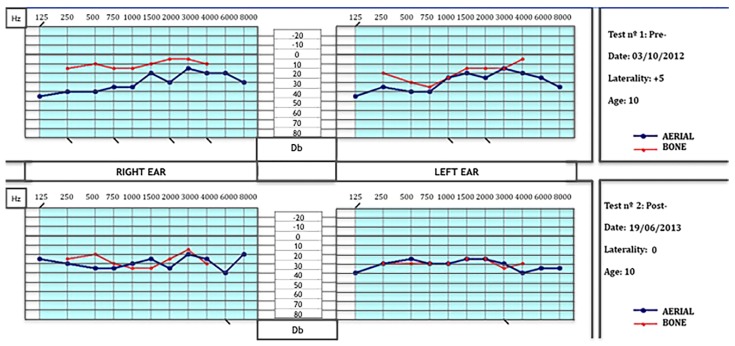
Aerial and bone auditive transmission pathways. **Upper**: Notice the low listening thresholds for all frequencies in both ears (especially for mids and lows) and the inverted curves of transmission (more in the right ear), and the spatialization errors for high frequencies. **Lower**: At the end of the treatment, curves of aerial and bone transmission pathways had been practically corrected and most of the spatialization errors had disappeared. (Db = decibels; Hz = Hertzs).

As Figure 1 shows (upper graphs, test nº 1), listening thresholds were relatively low for all frequencies in both ears, especially for mids and lows, which are frequencies related to language and the vestibular area, respectively. In addition, aerial and bone transmission pathways were completely inverted in both ears (more in the right ear) and there were spatialization errors and closed selectivity for high frequencies. In order to correct these abnormalities we designed a program, with filters for specific frequencies in the electronic ear, so that the frequencies listened to could work on different aspects: inter-hemispheric relationship, attention deficit, concentration, memory, learning, processing speed, spatial and temporal orientation, stimulation of the drivers of language, improving threshold listening, balance, coordination, verticality, reasoning, control of emotions and impulsivity.

As the lower graphs of Figure 1 show (test nº 2), listening thresholds still were low for the age of the patient, but the curves of the aerial and bone transmission pathways had been practically corrected and most of the spatialization errors had disappeared. This favored the improvements observed in language (the patient speaks coherently, she uses verb forms in past and future and not only the infinitive form as she did before), and the patient began to read, and clearly improved in memory, attention and concentration.

### 3.6. Blood Analysis

All blood tests were normal. IGF-I plasma values increased from 202 ng/mL at admission to 407 ng/mL at discharge (range for the age of the patient: 75–550 ng/mL). No adverse effects due to GH treatment were observed.

## 4. Discussion

Here we described the evolution of a patient with several cognitive deficits acquired after a significant birth asphyxia. PA is one recognized cause of cerebral palsy [3,4,5,11]; however, mild-moderate asphyxia has been associated with cognitive and behavioral alterations, such as hyperactivity, autism [4], attention deficits in children and adolescents [6,7], low IQ [8] (without significant motor impairments), and these are just the problems that affected our patient.

The patient had received intense neurorehabilitation over the years without significant improvements; in our experience, one of the reasons why traditional neurorehabilitation measures do not have significant effects on an injured brain is due to the fact that affected children usually begin to receive neurorehabilitation at ages in which the functional development of the brain has ended. Moreover, early care usually means 30–45 min of stimulation once (twice in very few cases) per week. Therefore, it is likely that GH administration played an important role, helping the neurorehabilitation performed, on the positive and quick results obtained in this case, despite the fact that the patient had a normal GH secretion. Furthermore, if GH had been administered at an earlier age, together with neurorehabilitation, it is likely that earlier and better results would have been obtained, according to our previous data in a child who suffered a cardiac arrest at birth of 20 min [28] and, as a consequence of it, experienced severe brain damage that was fully recovered one year later (GH treatment commenced at the age of one month in this case).

GH is a pleiotropic hormone expressed not only in the pituitary but in almost any tissue [30]. Thus, far beyond its classical actions on body growth and intermediate metabolism, GH exerts an important role in the regulation of cell proliferation and survival in several tissues, including the central nervous system (CNS) [31,32,33,34].

The hypothesis that GH and IGF-I play a role on brain repair after an injury was postulated years ago. GH expression within the CNS has been reported by several authors; however, its physiological role and, in particular, its contribution to the reparation of neurologic injuries remain poorly understood despite the positive effects that have been demonstrated in laboratory animals with GH treatment on adult neurogenesis [34,35,36,37], and recent data from our group and other groups [16,17,38,39] suggest that the hormone may play a similar role in non-GHD human patients [18,19]. In this regard, it is remarkable that the patient presented a frontal lobe malacia together with other brain sequelae produced by the asphyxia suffered at delivery.

As described in the Introduction, the GH/IGF-I axis plays a pivotal role during human fetal brain development and a very important role on cognitive functions afterwards [25]. In fact, the hormone and its receptor are expressed in the neural progenitor cells that exist in a number of cerebral neurogenic niches, inducing the proliferation, differentiation, migration and survival of these cells and newly-formed neurons [40]. The hormone induces the expression of IGF-I and its receptor in hippocampal human neural progenitors [41]. In rats, GH administration may cooperate with locally produced GH in brain repair after an injury [37] and peripheral GH induces cell proliferation in the intact adult rat brain [42]. Moreover, GH facilitates brain plasticity [26]; the hormone induces the expression of a number of neurotrophic factors and increases the cerebral metabolic turnover of neurotransmitters such as NA and DA [27].

Given the neurogenic actions of GH it is feasible to expect correlations between the GH status and learning and memory abilities (for a detailed review see references [25,43]): these are the deficits that our patient presented which were corrected with GH administration and neurorehabilitation. Moreover, a recent study in injured rats [44] demonstrated that GH treatment had a positive effect on cognitive function, most likely by increasing expression of hippocampal and prefrontal BDNF and TrkB.

## 5. Conclusions

In summary, we demonstrated here that GH therapy added to neurorehabilitation may play a significant role in the recovery of cognitive functions after brain damage, even though the patient is non-GHD. This agrees with previous postulates from our group and others [18,19,38,39,43]; however, since it is difficult to define what percentage of the positive effects shown here are due to GH administration or to the neurorehabilitation performed or both, a controlled trial will be necessary to produce stronger results in favor of GH plus neurorehabilitation treatment, as this is only a case report without any controls other than the patient.
